# Development of borated mesoporous zirconia nanocatalyst for the green synthesis of hydroquinone diacetate

**DOI:** 10.1038/s41598-025-27417-8

**Published:** 2025-11-26

**Authors:** Amr A. Ibrahim, Youstina S. Messiha, S. A. El-Hakam, Reda S. Salama, Awad I. Ahmed

**Affiliations:** 1 Chemistry Department, Faculty of science, Mansoura University, Mansoura, Egypt; 2 Basic Science Department, Faculty of Engineering, Delta University for Science and Technology, Gamasa, Egypt

**Keywords:** Mesoporous zirconia, Boric acid, 1,4-diacetoxybenzene, Catalysis, Heterogeneous catalysts, Solid-acid catalysts, Catalysis, Materials chemistry, Chemical synthesis

## Abstract

The demand for highly active and reusable solid acid catalysts has led to the development of borated mesoporous zirconia nanoparticles, which exhibit excellent catalytic performance in the green synthesis of pharmaceutical intermediates. This study focuses on the preparation, characterization, and catalytic application of borated mesoporous zirconia for the efficient production of 1,4-diacetoxybenzene, a valuable compounds in drug synthesis. The catalyst was synthesized using a sol-gel method, followed by boric acid functionalization to enhance surface acidity and surface area. Comprehensive characterization through XRD, FTIR, TEM, BET, and Thermal analysis confirmed the successful formation of a highly porous, thermally stable, and well-dispersed boron-modified zirconia structure. The catalytic efficiency was evaluated in the acetylation of hydroquinone under optimized conditions, where a maximum yield of 95.7% was achieved at 80 °C within 90 min using a catalyst loading of 25 wt%. Compared to traditional homogeneous acid catalysts, borated mesoporous zirconia exhibited superior stability and recyclability, as demonstrated by its ability to maintain 78% of its initial activity after three consecutive reaction cycles, with only a 18% decline in efficiency. The heterogeneous nature of the catalyst facilitated its easy recovery and reuse, reducing waste generation and operational costs. The results of this study highlight the potential of borated mesoporous zirconia as a sustainable, cost-effective, and environmentally benign catalyst for organic synthesis, offering significant advantages in pharmaceutical and fine chemical industries.

## Introduction

Preparation of both highly active and heterogeneous catalysts with reasonable recyclability could extremely eliminate harsh workups and therefore minimize the consumption of unnecessary substances, energy, and time required. From the standpoint of environmentally benign organic synthesis, the design of novel, highly active, and reusable immobilized catalysts has become a major area of research^1,2^. Various materials such as inorganic solids, polymers, and ionic liquids have been explored as supports, offering economic and environmental benefits^3^. Over the past 20 years, scientists have concentrated on sustainable chemistry due to the growing number of pollutants in the environment. The primary goal of sustainable chemistry is to minimize waste by using solvent-free or green solvents, low-temperature alterations, and reusable solid catalysts in place of liquid homogeneous catalysts to create a chemical pathway that is both economical and environmentally friendly^4–7^. The development of solid catalysts has been the subject of extensive investigation. Mesoporous materials have created new opportunities for use in drug delivery, separation science, protein encapsulation, catalysis, and catalyst supports due to their large surface areas, ease of functionalization, and controlled pore sizes^5,8^. Many scholars have been interested in mesoporous structures and preparation techniques over the last few decades^9–12^.

Due to their easily adjustable pore structures and surface characteristics, mesoporous metal oxides (MMO) are of great interest^13^. Numerous mesoporous materials, including doped materials with unique pore structures and chemical compositions, have been produced through the controlled synthesis of mesoporous metal oxides^14^. Moreover, distinct reactive sites are induced on the heterogeneous catalyst surface by the structural characteristics of MMOs. For instance, adding a second metal facilitates the generation of acidic sites on the catalyst, making it suitable for specific catalytic applications^15–17^. One significant class of advanced oxide is zirconia^18,19^, which has been extensively studied due to its unique properties, including ionic conductivity, reflectivity, compressive strength, and chemical stability^20^. Zirconia’s unique properties make it useful in a wide range of applications, including solid fuel cells, ceramics, thermal barrier coatings, catalysts, pigments, and luminescent materials or sensors when doped with rare earth elements^21–27^. In addition to zirconia’s structural characteristics, including crystallinity, particle size, and morphologies, its porosity plays a crucial role in determining its performance for specific applications.

There has been significant interest in developing mesoporous zirconia with a narrow pore size distribution for particular uses. The sol–gel method, utilizing ammonium salts as surfactants with linear alkyl chains, has been employed to create mesoporous zirconia, where the porous texture is influenced by different parameters^28,29^. A sol–gel mineralization method employing nonionic alkyl-PEO surfactants and hydrothermal treatment has produced mesoporous zirconia with a macroporous hierarchical funnel-like structure^30,31^. Recently, a precipitation method using ethylenediamine as a precipitating agent and zirconyl chloride as a zirconium precursor, followed by hydrothermal treatment, was reported for developing nanocrystalline mesoporous tetragonal zirconia with a high surface area^32–34^. The development of sulfated zirconium oxide with ordered mesoporous channels for applications in heterogeneous catalysis has also been investigated^9,35^. Additionally, studies have explored the use of stabilizers and pore formers to maintain mesoporous structures during heat treatment^36,37^.

The incorporation of borate species into mesoporous zirconia has attracted significant attention due to its ability to enhance catalytic performance, particularly in acid-catalyzed reactions. Borated zirconia catalysts offer strong Brønsted and Lewis acidity, which is essential for promoting a variety of organic transformations. The modification process typically involves introducing borate groups via impregnation or sol–gel synthesis, leading to structural stabilization and improved acid site distribution. Furthermore, boron incorporation can influence the textural properties of zirconia, including increased surface area, controlled pore size, and enhanced thermal stability, making it a highly efficient solid acid catalyst^38,39^. Several studies have demonstrated the superior activity of borated mesoporous zirconia catalysts in esterification, alkylation, and dehydration reactions due to their improved acidity and stability^40–42^. These attributes make borated mesoporous zirconia an excellent candidate for green and sustainable catalytic applications.

Hydroquinone diacetate (1,4-diacetoxybenzene) is an important intermediate in the synthesis of various pharmaceuticals, agrochemicals, and fine chemicals. Its production via eco-friendly catalytic pathways is crucial for sustainable chemistry^43,44^. Traditional methods for its synthesis often involve homogeneous acid catalysts, which generate significant waste and pose separation challenges. The use of heterogeneous solid acid catalysts, such as borated mesoporous zirconia, provides an environmentally friendly alternative by enabling high yields, easy separation, and catalyst reusability^45^.

The present study focuses on the development of a borated mesoporous zirconia solid acid catalyst for the high-yield and environmentally friendly synthesis of drug-related 1,4-diacetoxybenzene. This research aims to explore the structural, textural, and catalytic properties of the synthesized catalyst and its efficiency in organic transformations. The study involves the synthesis and characterization of borated mesoporous zirconia using advanced techniques such as X-ray diffraction (XRD), scanning electron microscopy (SEM), nitrogen adsorption-desorption analysis, and Fourier transform infrared spectroscopy (FTIR). The catalytic performance is evaluated in the synthesis 1,4-diacetoxybenzene under optimized reaction conditions. The recyclability and stability of the catalyst are also investigated to determine its practical applicability in sustainable organic synthesis. By developing an efficient and reusable catalyst, this study contributes to the advancement of green chemistry and the reduction of environmental pollution associated with conventional catalytic processes.

## Experimental

### Materials

Cetyltrimethylammonium bromide (CTAB, purity ≥ 99%), zirconium oxychloride octahydrate (ZrOCl₂·8 H₂O, purity ≥ 99%), boric acid (H₃BO₃, purity ≥ 99%), ammonium hydroxide (NH₄OH, purity ≥ 99%), and silver nitrate (AgNO₃, purity ≥ 99%) were purchased from Sigma-Aldrich. All chemicals were used as received without further purification.

#### Synthesis of mesoporous zirconia

A mesoporous zirconia support was synthesized using a modified surfactant-assisted sol–gel method. In a typical synthesis, 3 g of cetyltrimethylammonium bromide (CTAB) was dissolved in 25 mL of distilled water with stirring until a homogeneous solution was achieved. Then, 2 mL of ammonium hydroxide (NH₄OH) was added dropwise to the CTAB solution under continuous stirring to adjust the pH to 8. Subsequently, 13 g of zirconium oxychloride octahydrate (ZrOCl₂·8 H₂O) was dissolved in 25 mL of distilled water and added dropwise to the CTAB solution, forming a white suspension. The resulting precipitate was stirred for 3 h at room temperature, then left overnight to settle. The mother liquor was decanted, and the precipitate was filtered and washed several times with distilled water to remove chloride ions, confirmed by AgNO₃ testing. The filtered cake was dried at 120 °C for 12 h, then calcined at 550 °C for 6 h to obtain pure mesoporous zirconium oxide (denoted as Z).

#### Modification of mesoporous zirconia with boron

To introduce boron into the mesoporous zirconia, the as-synthesized Z was impregnated with an aqueous solution of boric acid (H₃BO₃) to obtain different boron concentrations (5, 15, 25, 35, and 50 wt%). In a typical procedure, an appropriate amount of boric acid was dissolved in distilled water, and the Z support was added to the solution. The mixture was stirred for 4 h at room temperature to ensure uniform impregnation. Afterward, the solution was evaporated to remove excess water, and the resulting solid was dried in an oven at 120 °C overnight. The dried materials were then calcined at 400 °C (denoted as xBZ-I), 500 °C (xBZ-II), and 600 °C (xBZ-III) for 5 h. The number “I,” “II,” and “III” after Z indicates the calcination temperatures of 400, 500, and 600 °C, while the letter “x” represent the boron content in weight% (e.g., 5BZ-I for 5 wt% boron, calcined at 400 °C).

### Characterization

The thermal properties of the prepared composites were analyzed by thermogravimetric (TGA) and differential thermal analysis (DTA) using a Shimadzu thermal analyzer, model 50-H. The samples were heated in a nitrogen stream (20 mL/min) at a heating rate of 10 °C/min, with sintered alumina (α-Al₂O₃) used as the thermally inert reference material. Temperature measurements were conducted using a Pt/Rh (10%) thermocouple. X-ray diffraction (XRD) analysis was performed on the catalysts using a Philips PW 105 diffractometer with Ni-filtered Cu Kα radiation (λ = 1.540 Å) at an operating voltage of 40 kV and current of 30 mA. All samples were scanned at a speed of 2°/min from 2θ = 5° to 80°. Transmission electron microscopy (TEM) was employed to examine the morphology and particle size of the catalysts. The micrographs were captured using a Gtan slow-scan charge-coupled device (CCD) in a Phillips CM120 Biotwin electron microscope, operated at 120 kV. For the analysis, the sample was ultrasonically dispersed in ethanol, and a drop of the suspension was placed onto a holey carbon film supported on a copper grid. The functional groups and chemical bonding in the mesoporous materials were analyzed by Fourier transform infrared (FTIR) spectroscopy using a Nicolet Magna-IR 550 spectrometer. The spectra were recorded in the mid-IR range (400–4000 cm⁻¹) with a resolution of 4 cm⁻¹ and 128 scans, and the sample was prepared by grinding it with KBr and pressing it into a thin wafer. Finally, the textural properties of the catalysts, including surface area, pore size distribution, and pore volume, were determined by nitrogen adsorption isotherms at its boiling point of -196 °C, using a conventional volumetric apparatus. The total acidity of the catalysts was determined using potentiometric titration with the n-butylamine method, where 0.1 g of catalyst was heated under vacuum and mixed with 10 mL of acetonitrile. After agitation for 2 h, the suspension was titrated with 0.01 N n-butylamine in acetonitrile using an Orion 420 digital A model with a double junction electrode^46^. To further investigate the nature of the acid sites, pyridine adsorption experiments were performed to determine the Lewis and Brønsted acid site ratio. For this, 0.1 g of each sample was degassed at 250 °C for 2 h to remove adsorbed molecular water. The samples were then exposed to pyridine vapor at room temperature for 1 h^47^. The FT-IR spectra were recorded using a Mattsoh 5000 FT-IR spectrophotometer by mixing 0.01 g of the sample with 0.1 g of KBr to form 30 mm diameter self-supporting discs.

### Catalytic activity

#### Synthesis of hydroquinone diacetate

The catalytic efficiency was further assessed in the synthesis of hydroquinone diacetate. In a typical reaction, hydroquinone (1.1 g, 1.0 mol) and acetic anhydride (1.9 mL, 2.0 mol) were added to a 50 mL round-bottom flask equipped with a magnetic stirrer and a reflux condenser, in the presence of 0.015 g of the activated catalyst (25 wt% relative to hydroquinone). The reaction was conducted at 80 °C under solvent-free conditions and stirred for 90 min. The acetylation proceeded exothermically, and after completion, the reaction mixture was cooled to room temperature and poured into a 100 mL beaker containing crushed ice. The resulting white crystalline product was collected by vacuum filtration, washed with cold water, and purified by recrystallization from dilute ethanol. The purified product was identified as 1,4-diacetoxybenzene based on its melting point (121–122 °C), consistent with literature data. The product yield was determined gravimetrically using Eq. (1), and each reaction was repeated three times under identical conditions to confirm reproducibility, with observed variations within ± 2%.

1 $$\:yield\:\%=\frac{obtained\:weight\:of\:product}{theoritical\:weight\:of\:product}\times\:100$$

## Results and discussion

### Thermal analysis

**Fig. 1:**
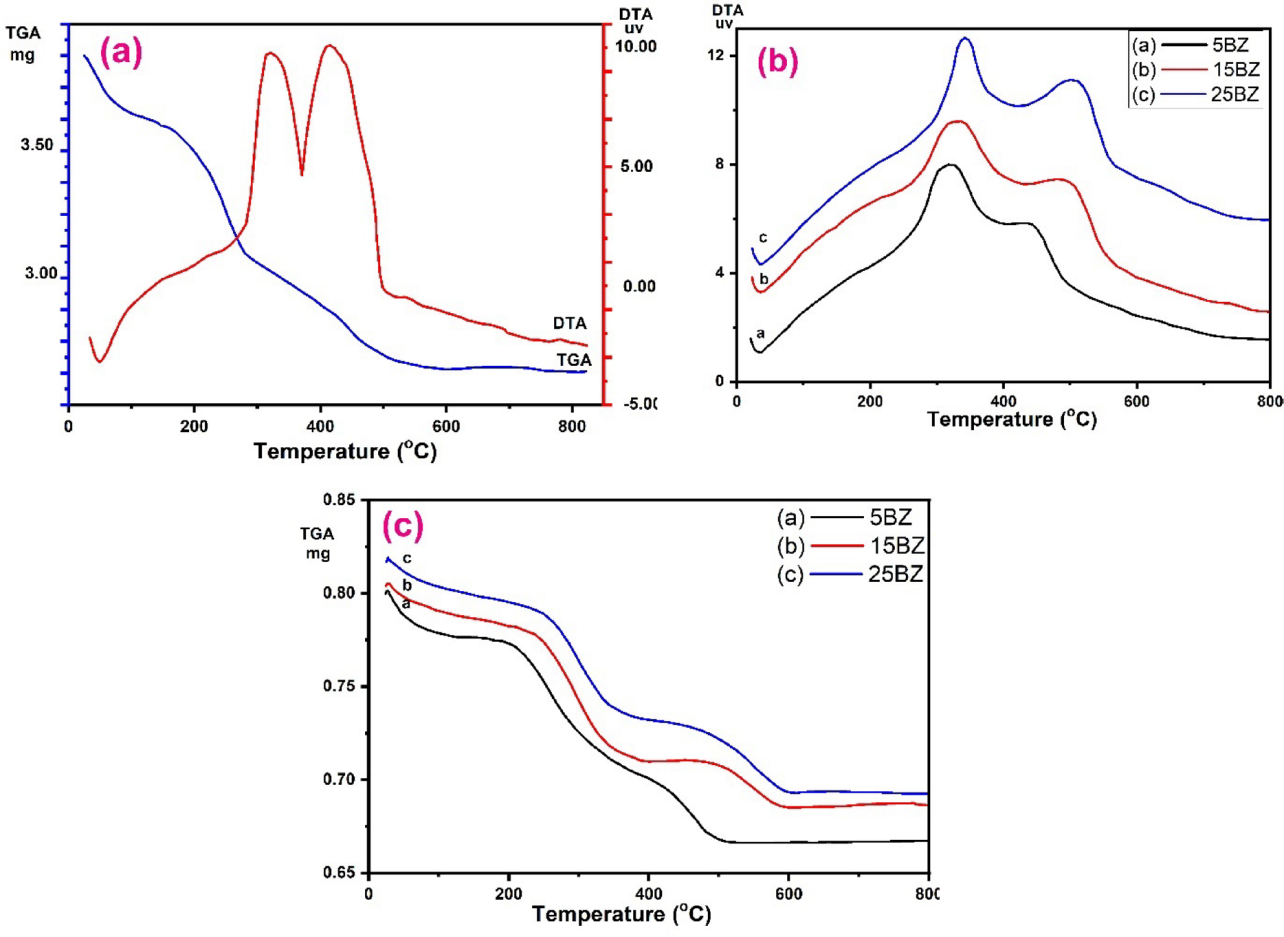
(**a**) DTA and TGA for Zr(OH)4 sample, (**b**) DTA, and (**c**) is TGA for different weight percentage borate on mesoporous Zirconia (5, 15, and 25wt.%).

Thermal analysis is a crucial technique to investigate the stability, phase transformations, and decomposition behaviour of zirconia and borated zirconia. The thermogravimetric analysis (TGA) provides insight into weight loss patterns, while differential thermal analysis (DTA) identifies the associated thermal events. Figure 1a presents a comparison of the DTA and TGA profiles of dried Zr(OH)₄, which exhibits a single endothermic peak at lower temperatures, corresponding to a weight loss attributed to the removal of physically adsorbed water^4^. As the temperature increases, two prominent exothermic peaks appear. The first peak, observed around 330 °C, is accompanied by a weight loss of approximately 9.3%, which can be associated with the dehydroxylation process and the thermal decomposition of the surfactant template^48,49^. The second peak at approximately 425 °C, with a corresponding weight loss of 4.3%, is indicative of the transformation of zirconium oxyhydroxide into crystalline ZrO₂^50^. The TGA curves illustrate the thermal stability and decomposition behaviour of zirconia (5BZ) and borated zirconia (15BZ and 25BZ) were shown in Fig. 1b,c. The weight loss in all samples occurs in multiple stages. Initially, a minor weight loss below 150 °C is observed in all samples due to the desorption of physically adsorbed water and moisture^51^. This is followed by an intermediate weight loss between 150 and 400 °C, which corresponds to the decomposition of residual organic species and structural water from the zirconia matrix^52^. The borated samples (15BZ and 25BZ) exhibit slightly lower weight loss, indicating enhanced thermal stability due to borate incorporation. At higher temperatures (400–800 °C), the weight loss stabilizes, suggesting the formation of thermally stable zirconia and borated zirconia phases. The negligible weight loss in this region highlights the refractory nature of the materials. The DTA curves provide additional insights into the thermal events occurring during heating. An endothermic event around 100–150 °C corresponds to the evaporation of physically bound water. Exothermic peaks observed between 250 and 400 °C are more prominent in borated zirconia samples and indicate phase changes or crystallization processes. The presence of borate influences the crystallization behaviour, shifting the exothermic peaks slightly. At higher temperatures above 400 °C, broad exothermic peaks suggest the formation of stabilized zirconia phases. The incorporation of borate modifies the phase transition temperatures, reflecting its interaction with the zirconia framework. The TGA/DTA analysis reveals that borated zirconia exhibits improved thermal stability compared to pure zirconia. The incorporation of borate reduces weight loss and alters the phase transformation temperatures, which can be beneficial for catalytic applications. The enhanced stability suggests that borated zirconia can be a promising material for high-temperature catalytic reactions and other energy-related applications.

### X-ray diffraction analysis

**Fig. 2:**
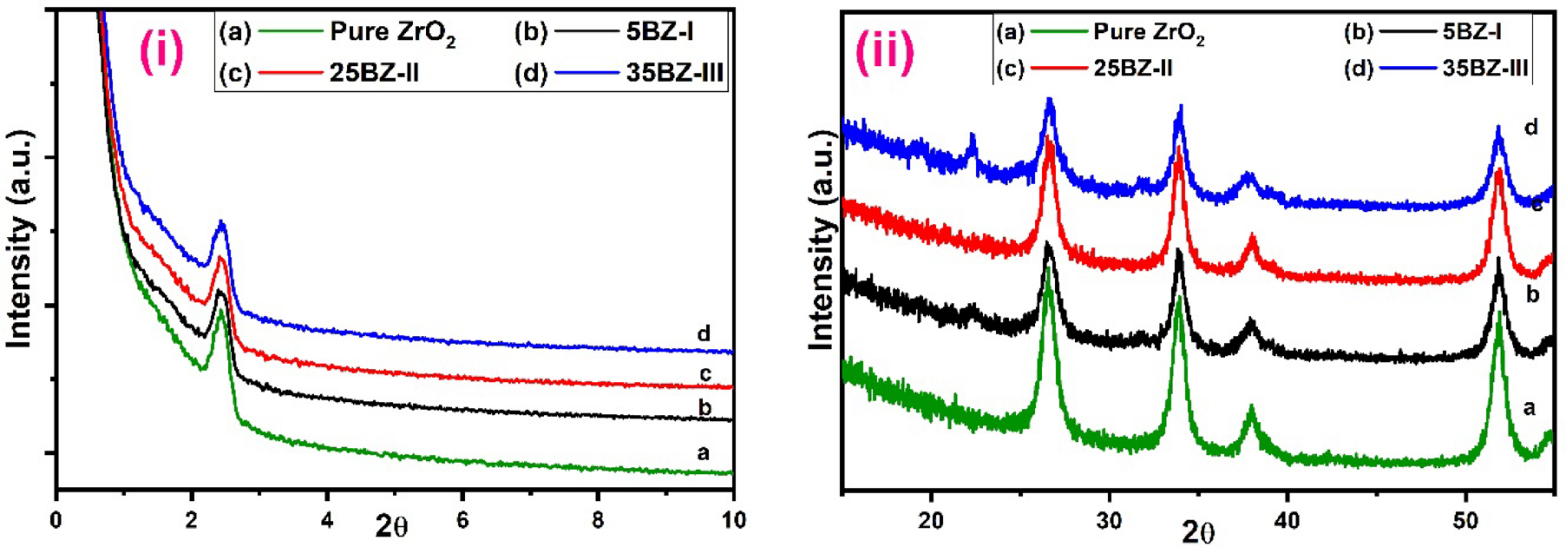
(**i**) Low and (**ii**) Wide angle XRD patterns of the sample (a) ZrO2, (b) 5BZ-I, (c) 25BZ-II and (d) 35BZ-III.

The X-ray diffraction (XRD) analysis was conducted to investigate the crystalline phases, structural modifications, and the effect of borate incorporation on zirconia. The XRD patterns of pure zirconia and borated zirconia samples at different compositions are presented in Fig. 2i and ii. The diffractograms reveal distinct features corresponding to the amorphous and crystalline nature of the materials. Figure 2i shows the low-angle X-ray powder diffraction patterns of mesoporous ZrO_2_ sample calcined at 550 °C and modified borated ZrO_2_. The peak in low angle at 2θ = 2.4° corresponds to the (100) reflection, which is assigned to the presence of hexagonal mesostructured material^53^. The addition of borate and increasing calcination temperature (Fig. 2i(b-d)) show no effect on the hexagonal mesostructured ZrO_2_, emphasizing that the catalysts still retain their mesostructured even with increasing borate content. The intensity of this peak varies with increasing borate content, suggesting structural modifications due to borate incorporation. The increase in intensity with higher borate loading implies enhanced disorder and a possible change in pore structure within the zirconia framework. Figure 2ii(a) shows wide angle XRD patterns of mesoporous ZrO_2_ calcined at 550 °C, and the peaks agree well with the monoclinic crystalline structure of mesoporous ZrO_2_. The monoclinic phase lines were located at 2θ = 26.52°, 33.89°, 38.11°, and 51.87°, corresponding to (110), (101), (200), and (211) reflection monoclinic planes of mesoporous ZrO_2_, respectively, as given in the standard data file (JCPDS No. 41-1445)^54^. The XRD patterns of BZ-I at different BO_3_^3−^ content, 5, 25, and 35 wt% (Fig. 2ii(b-d)), showed patterns similar to those of the pure mesoporous ZrO_2_ in monoclinic form, and the degree of crystallization gradually decreased with increasing BO_3_^3−^ content, while the width of the reflections considerably broadened, indicating a small crystalline domain size. A comparison of the XRD patterns of different borated zirconia samples reveals that higher borate concentrations lead to an increase in peak broadening, suggesting a reduction in crystallite size. This observation aligns with the influence of borate in stabilizing ZrO_2_ phase and preventing grain growth^55^. Additionally, no additional peaks corresponding to separate borate phases are detected, indicating that borate is well-dispersed within the zirconia matrix without forming distinct crystalline phases.

**Fig. 3:**
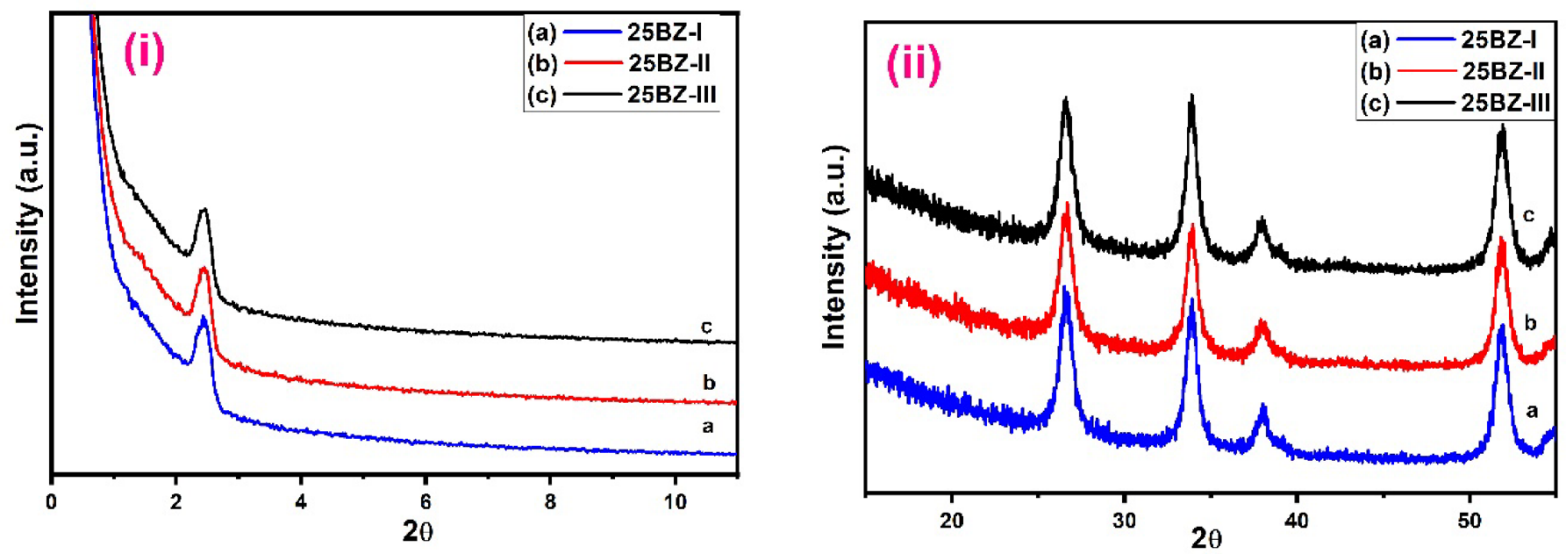
(**i**) Low and (**ii**) Wide angle XRD patterns of 25BZ at different calcination temperature (a) 400 °C, (b) 500 °C, and (c) 600 °C.

The effect of calcination temperature on the crystalline structure of borated zirconia was also analyzed and displayed in Fig. 3i and iii. As the calcination temperature increased, a gradual transformation from an amorphous to a more crystalline structure was observed. At lower temperatures, the XRD patterns exhibited broader peaks, indicating smaller crystallite sizes and less defined crystalline phases. However, with increasing temperature, the peaks became sharper and more intense, suggesting enhanced crystallization and grain growth. The thermal energy provided during calcination facilitates atomic rearrangement, leading to improved ordering of the zirconia lattice^56^. Additionally, a minor shift in peak positions was observed with increasing temperature, which may be attributed to lattice strain and changes in crystallite size. The crystallite sizes of mesoporous ZrO_2_ and BZ samples were calculated from the broadening of the strongest peak of mesoporous ZrO_2_ at 2θ = 26.52° based on Scherrer’s equation. The crystallite size confirmed that higher calcination temperatures led to an increase in grain size, which is expected due to the combination of smaller particles into larger ones at elevated temperatures. Despite these changes, the presence of borate inhibited excessive grain growth, maintaining the mesoporous structure and enhancing the stability of the material. These findings highlight the crucial role of calcination temperature in determining the structural properties and stability of borated zirconia. Examination of Table 1 shows that the crystallite size of pure mesoporous ZrO_2_ decreases as the BO_3_^3−^ content increases, and the crystallite size increases gradually with increasing calcination temperature up to 600 °C. Also, the change in values of d_100_-spacing and a_o_ by increasing BO_3_^3−^ content and calcination temperature is very small, keeping borated zirconia samples in their porous structure. It was reported that the mesoporous order of mesostructured materials strongly depends on the texture properties of the pores. The crystallite sizes of the samples suggests that pure zirconia has a relatively larger crystallite size compared to borated zirconia samples. The decrease in crystallite size with increasing borate content further supports the hypothesis that borate inhibits crystallization and stabilizes smaller zirconia domains. This effect is particularly significant for samples with higher borate concentrations, where the broadening of peaks becomes more pronounced. Overall, the XRD analysis confirms that borate incorporation leads to structural modifications in zirconia by enhancing amorphization, inducing lattice distortion, and reducing crystallite size.

**Table 1:** Effect of BO_3_^3−^ content and calcination temperatures on crystallite size of ZrO_2_ catalysts.

Sample	Crystallite size (nm) By XRD	d_100_ (nm)	a_o_ (nm)	Crystallite size (nm) By TEM
ZrO_2_	28.95	3.14	3.70	28.3
5BZ-I	18.99	3.18	3.74	20.6
25BZ-I	17.41	3.19	3.75	17.6
35BZ-I	17.02	3.20	3.80	16.9
25BZ-II	18.67	3.18	3.74	18.6
25BZ-III	19.01	3.18	3.74	19.1

### FTIR spectra

**Fig. 4:**
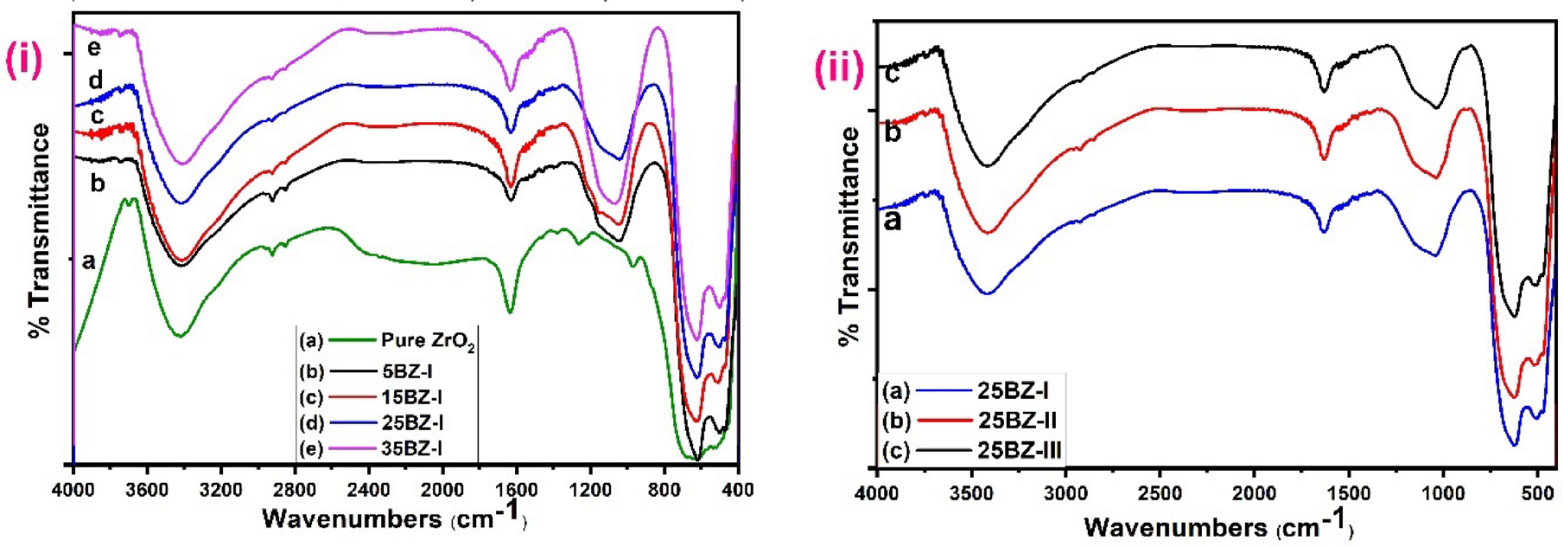
FTIR spectra of (**i**) pure and modified ZrO_2_ with different borate content and (**ii**) 25BZ at different calcination temperature.

The FTIR spectra of pure ZrO₂ and borated zirconia samples are presented in Fig. 4i, recorded in the 400–4000 cm⁻¹ range. The characteristic absorption bands of pure ZrO₂ appeared at 620, 1636, 2912, and 3406 cm⁻¹. The peak at 620 cm⁻¹ corresponds to Zr–O and Zr–O–Zr stretching vibrations, confirming the presence of zirconia^9^. The broad absorption band around 3406 cm⁻¹ and the peak at 1636 cm⁻¹ are associated with the stretching and bending vibrations of hydroxyl (-OH) groups, indicating surface-adsorbed water molecules^18^. The peak at 2912 cm⁻¹ is attributed to C–H stretching vibrations, likely due to residual organic species from the synthesis process^50^. Upon borate incorporation, the FTIR spectra of borated zirconia samples exhibit additional peaks at 479, 620, and 1085 cm⁻¹. The new band at 479 cm⁻¹ corresponds to the bending vibration of O–B–O bonds, confirming the presence of BO₃³⁻ units. The broad band at 1045 cm⁻¹, assigned to the stretching vibration of the B–O bond in BO₃³⁻, shifts to a higher frequency of around 1068 cm⁻¹ as borate loading increases to 25 wt%. This shift indicates enhanced covalency in the B–O bonds due to stronger electron interactions with the ZrO₂ framework^57^. Based on the observed absorption bands, the presence of borate species can be inferred, primarily in the form of trigonal BO₃ units; however, due to the limitations of FTIR alone, the specific identification of tetrahedral BO₄ units cannot be confirmed and has therefore been removed. Additionally, the broadening of the hydroxyl (-OH) bands at 3406 cm⁻¹ and 1636 cm⁻¹ suggests an increased number of surface hydroxyl groups, which may enhance the catalytic activity of borated zirconia by providing more active sites for adsorption and reaction. On the other hands, FTIR spectra of borated zirconia (25BZ) calcined at different temperatures (Fig. 4ii) show no significant changes in peak positions or intensities, confirming that the BO₃³⁻ and BO₄ structures remain stable upon heating. The characteristic B–O stretching vibrations at ~ 1068 cm⁻¹ and the O–B–O bending at ~ 479 cm⁻¹ persist, indicating that the borate species are strongly anchored to the zirconia surface. This thermal stability suggests that borate-modified zirconia retains its mesostructured framework even at elevated temperatures.

### Texture properties of borated zirconia

**Fig. 5:**
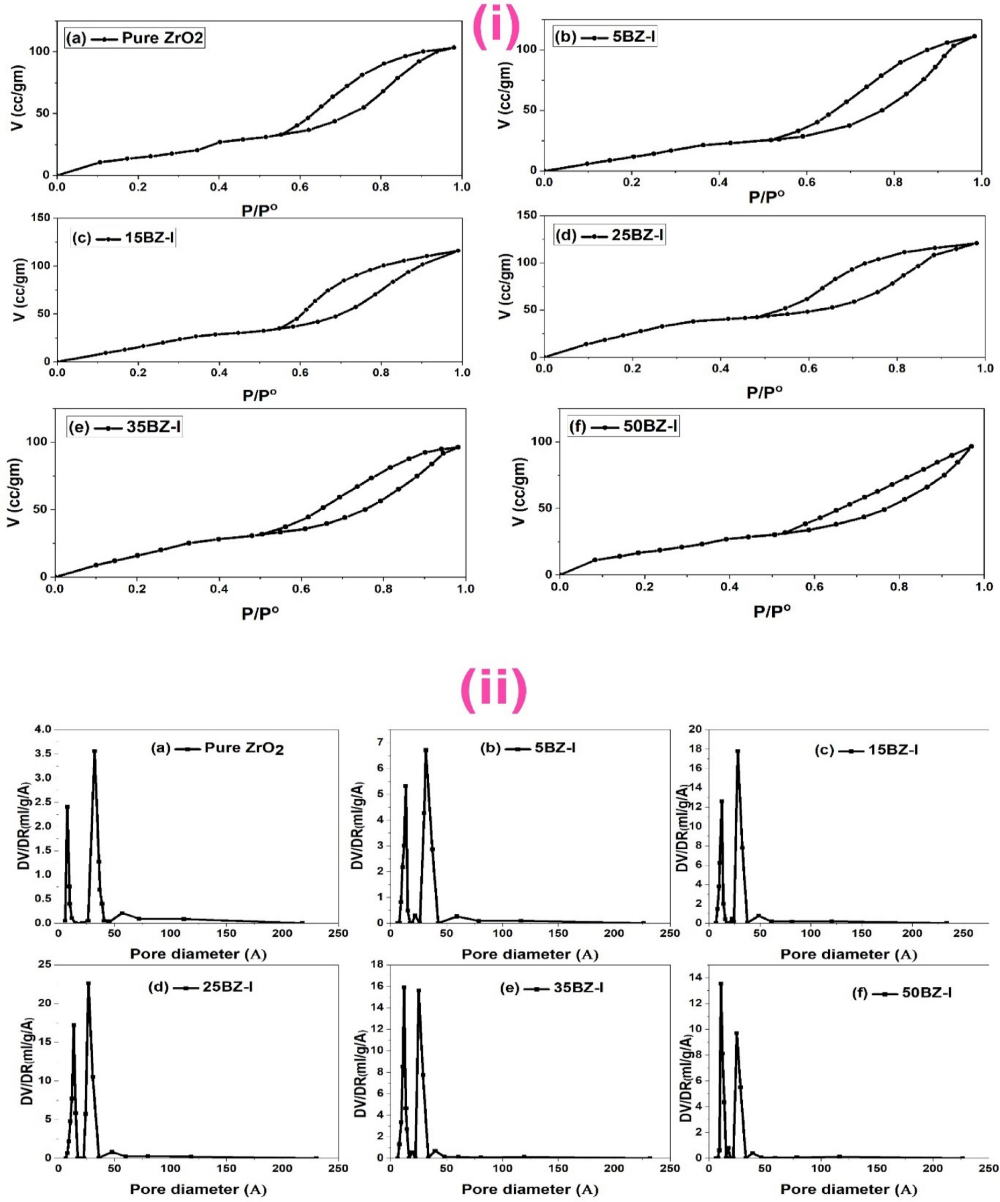
N_2_ sorption isotherms (**i**) and corresponding pore size distribution curves (**ii**) of Pure and modified ZrO_2_.

Figure 5i presents the N₂ adsorption-desorption isotherms of BZ-I at different borate loadings (5, 15, 25, 35, and 50 wt%), while Fig. 5ii illustrates the corresponding pore size distribution curves. The isotherms exhibit characteristic type IV behaviour with an H3 hysteresis loop, indicating the presence of mesoporous structures^58^. The adsorption capacity initially increases with the incorporation of BO₃³⁻ up to 25 wt%, reaching a maximum due to the improved dispersion of borate species on the ZrO₂ surface. This enhancement in adsorption capacity suggests that borate modifies the surface properties by preventing excessive crystallization of ZrO₂, thereby stabilizing the mesoporous framework^59^. The increase in surface area (S_BET_) up to 25 wt% loading, as shown in Table 2, supports this observation. However, further addition of BO₃³⁻ beyond this threshold leads to a decline in surface area, likely due to pore blocking effects caused by excess borate species^60^. The BJH pore size distribution (Fig. 5ii) confirms that with increasing borate content, the maximum peak shifts slightly towards smaller pore diameters, indicating partial occlusion of the mesopores. This phenomenon results from the deposition of additional borate layers, which progressively reduce the available pore volume (V_T_). The interplay between pore volume, pore diameter, and surface area highlights the role of borate in tuning the textural properties of ZrO₂.

**Fig. 6:**
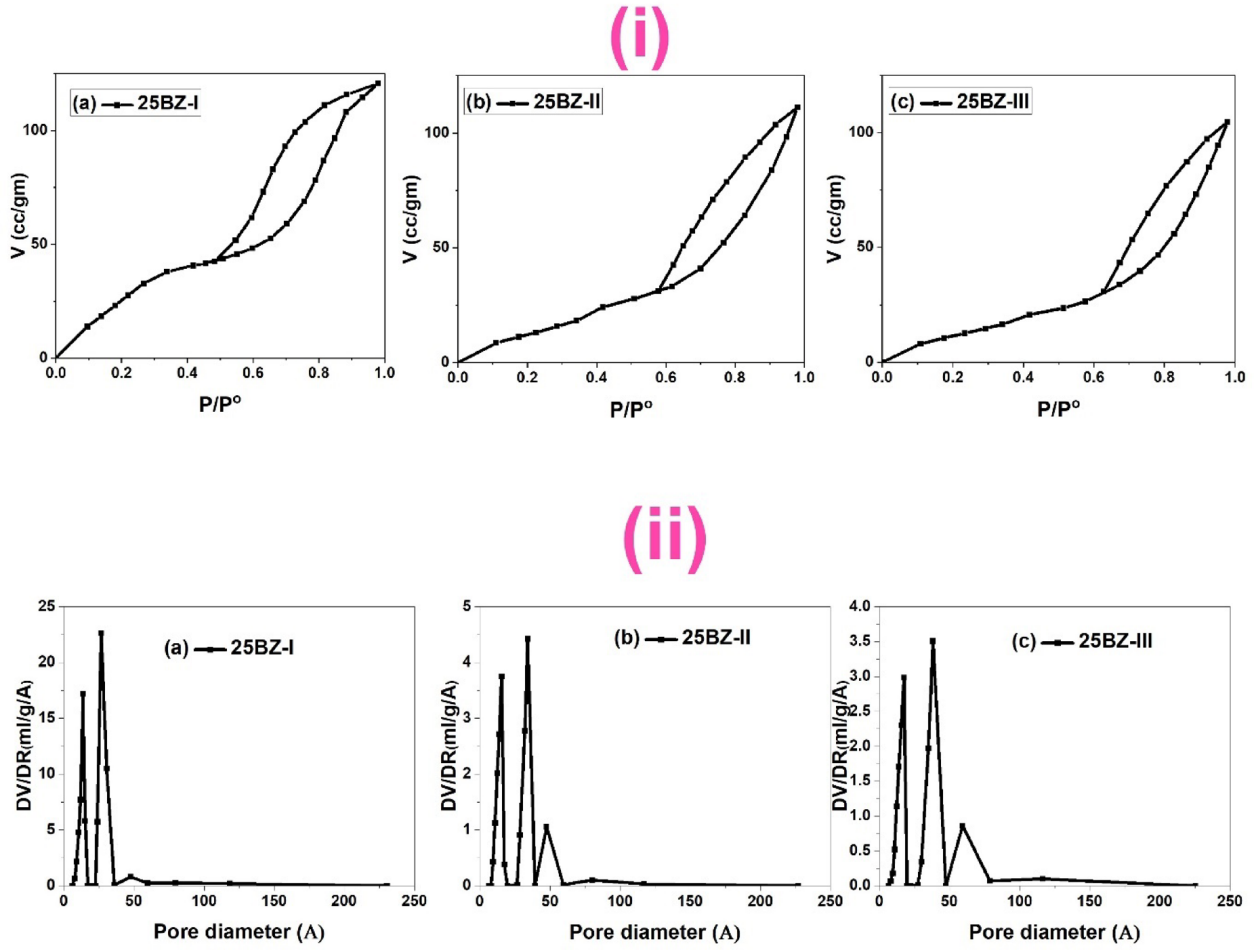
N_2_ sorption isotherms (**i**) and corresponding pore size distribution curves (**ii**) of 25BZ at different calcination Temperature (400, 500, and 600 °C).

The influence of calcination temperature on the porous characteristics of 25BZ is represented in Fig. 6i (N₂ adsorption-desorption isotherms) and Fig. 6ii (pore size distribution). The isotherms maintain the typical mesoporous profile; however, a noticeable decrease in nitrogen adsorption capacity is observed with increasing calcination temperature. This trend indicates progressive densification of the material, which leads to a decrease in available surface area (S_BET_), as displayed in Table 2. The surface area reduction can be attributed to pore collapse and sintering effects, where higher thermal treatment enhances crystallite growth, reducing the contribution of mesopores to the overall porosity. The total pore volume (V_T_) follows a similar downward trend, confirming that elevated calcination temperatures lead to the contraction of the porous network^61^. The BJH pore size distribution (Fig. 6ii) further supports this observation, showing a shift toward slightly larger pore diameters, which can be linked to the merging of smaller pores and partial coalescence of mesostructures. These modifications in textural properties align with the expectations of thermally treated mesoporous materials, where increased thermal energy promotes atomic diffusion and rearrangement. The decrease in microporosity and total pore volume suggests that optimal calcination conditions are crucial in preserving the mesostructure of borated ZrO₂. The combination of 25 wt% borate and 400 °C calcination strikes a balance between structural stability and textural integrity, ensuring an ideal porous architecture for catalytic applications. These findings reinforce the significance of controlled thermal treatment in fine-tuning the porosity and surface characteristics of borated zirconia-based catalysts.

### Surface morphology of the prepared sample

**Fig. 7:**
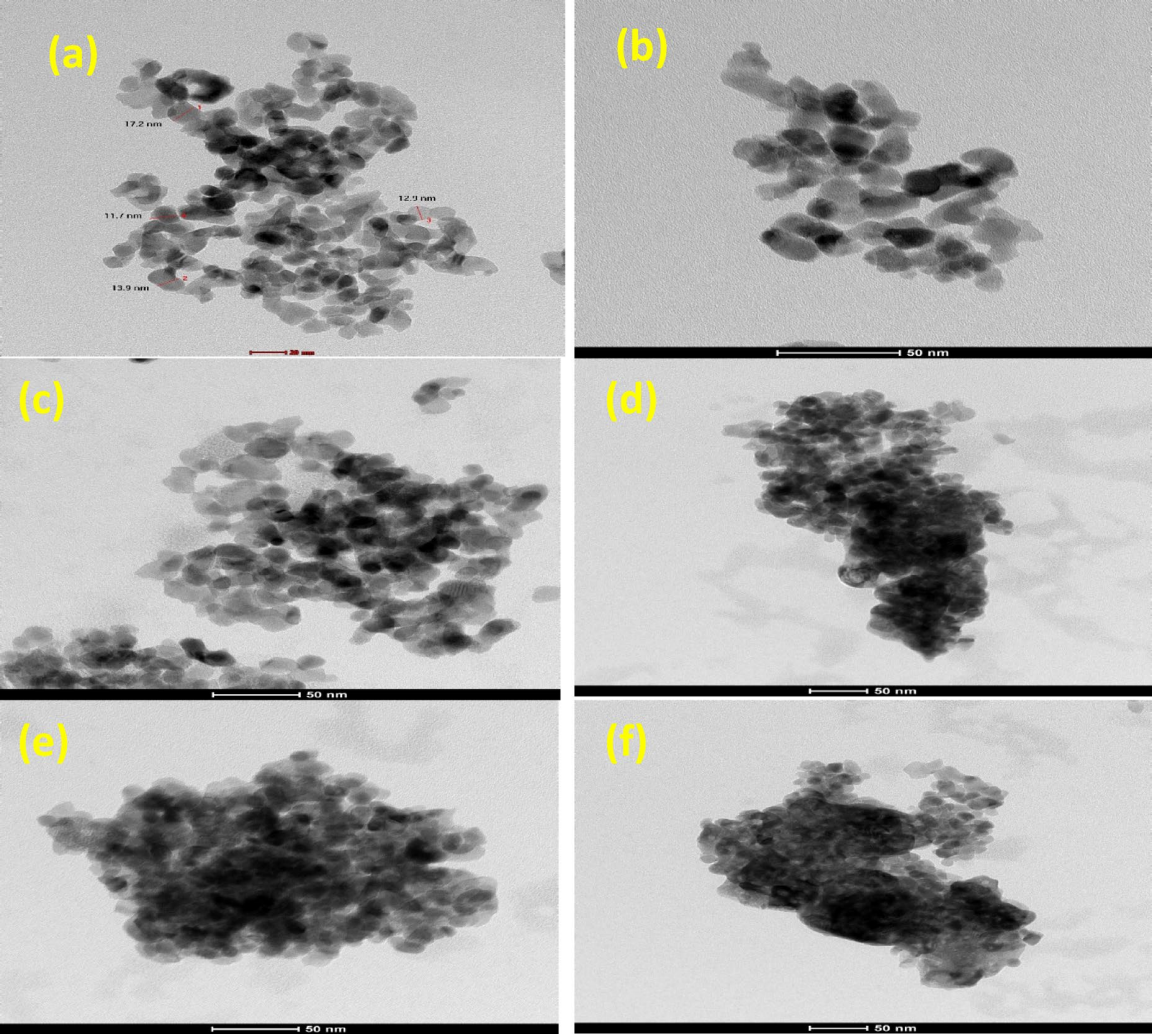
TEM images of (**a**) pure ZrO_2_, (**b**) 5BZ-I, (**c**) 25BZ-I, (**d**) 35BZ-I, (**e**) 25BZ-II, and (**f**) 25BZ-III.

**Fig. 8:**
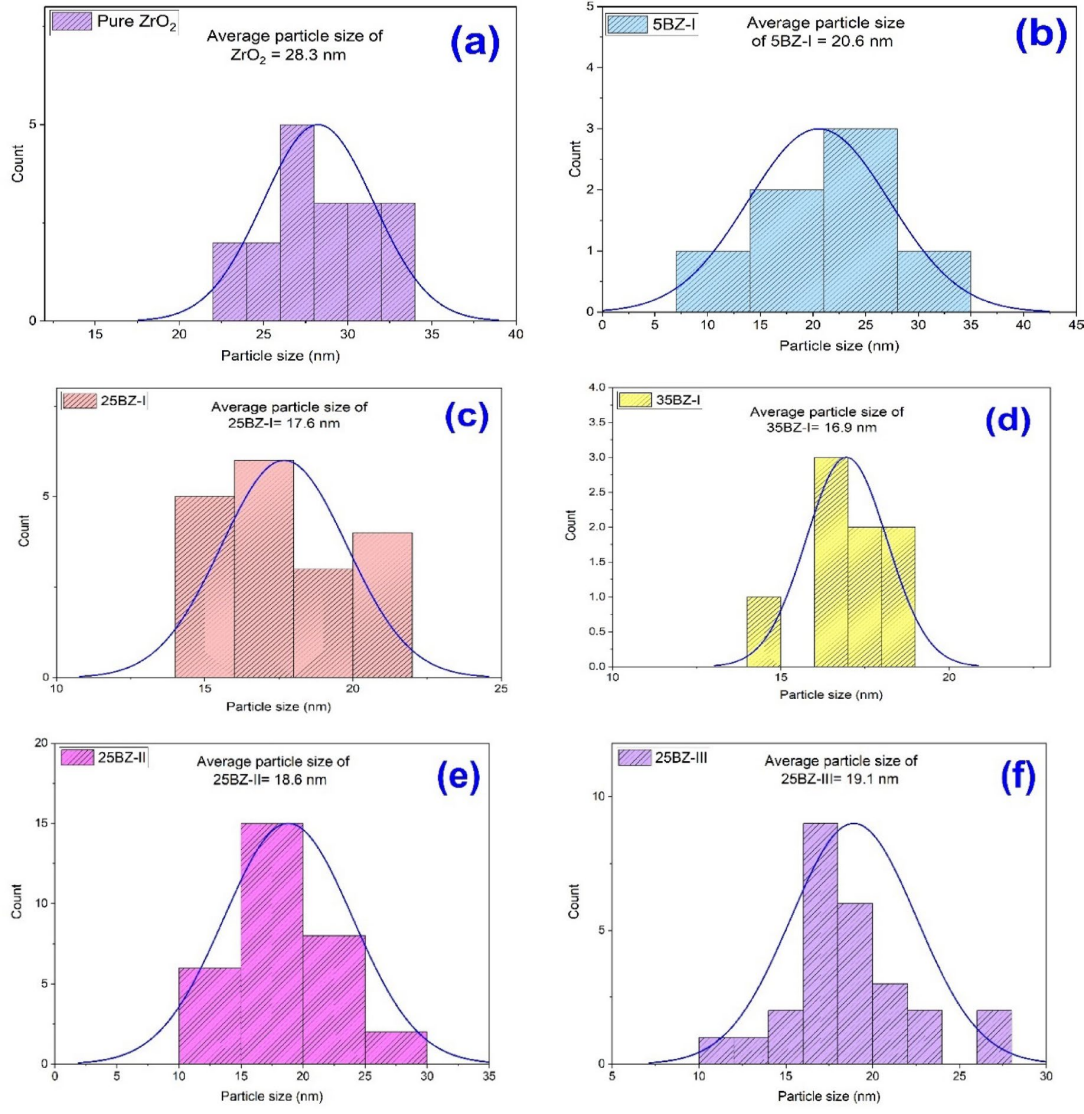
Particle size distribution of (**a**) pure ZrO_2_, (**b**) 5BZ-I, (**c**) 25BZ-I, (**d**) 35BZ-I, (**e**) 25BZ-II, and (**f**) 25BZ-III.

The transmission electron microscopy (TEM) images provide insightful structural information regarding the morphology, particle size distribution, and structural preservation of borated zirconia (BZ) samples. As observed in Fig. 7a, pure ZrO₂ exhibits a uniform, nanoscale particle distribution with no signs of dense agglomeration, suggesting a well-developed porous network. While distinct mesoporous channels are not clearly resolved in the TEM images—likely due to resolution limitations or overlapping structures—the nanoscale morphology and high dispersion indicate features consistent with mesoporous materials^62,63^. Upon borate loading, as displayed in Fig. 7b–d, the particle morphology remains consistent, indicating that BO₃³⁻ species are effectively embedded within the ZrO₂ framework without disrupting the overall structure. Although some distortion is observed at higher borate concentrations (e.g., 35BZ-I), the framework remains intact, suggesting partial pore blockage due to surface saturation. Furthermore, Fig. 7e and f show that even after calcination at 500 and 600 °C, the morphology is retained, highlighting the stabilizing effect of borate on thermal stability. The particle size distribution analysis further supports these findings, revealing particle sizes of 28.3 nm, 20.6 nm, 17.6 nm, and 16.9 nm for ZrO₂, 5BZ-I, 25BZ-I, and 35BZ-I, respectively, as displayed in Fig. 8; Table 1. These values correlate well with crystallite sizes determined by XRD, confirming the role of boron in inhibiting particle growth. Although mesopores are not directly observed in TEM, BET surface area analysis and low-angle XRD results clearly indicate the presence of mesoporous structures, validating the retention of porosity and confirming that borate modification effectively tunes the structural and textural properties of the material.

### Surface acidity of the investigated catalysts

#### Non-aqueous potentiometric Titration

The acidic properties of the prepared sampled were examined through non- aqueous titration^64^. This technique is a reliable method for assessing both the total acidity and acid strength of solid acid catalysts by measuring the electrode potential as the acid sites are progressively neutralized. As the titration proceeds, the curves exhibit an asymptotic trend, where a buffering effect becomes evident, and a plateau is reached, corresponding to the total number of acid sites (meq/g solid), that calculated using the next relation: ***Total number of acid sites / g =(mequiv./g) ×N×1000*** (Where N is Avogadro’s number)^10,65^. The amount of n-butylamine required for neutralization directly correlates with the surface acidity of the catalyst. As observed in Table 2, increasing the BA content leads to a gradual enhancement in both acid strength and total acidity, with the highest value recorded for 25BZ-I (Ei = + 520.2 mV). This suggests that the introduction of BA significantly contributes to the acidity of the catalyst by generating strong acid sites. However, when the calcination temperature is increased to 500 and 600 °C, a noticeable decline in total acidity is observed, as presented in Table 2. This reduction can be attributed to the thermal-induced agglomeration of BO_3_^3−^ species on the ZrO_2_ surface, which leads to a decrease in the number of accessible acid sites. The highest surface acidity at 400 °C indicates that this is the optimal calcination temperature, as it ensures a strong interaction between BO_3_^3−^ and the ZrO_2_ surface while preventing excessive sintering or structural collapse. The decline in acidity at elevated calcination temperatures suggests that prolonged exposure to high thermal conditions may cause the partial decomposition or transformation of active acid sites, thereby reducing the catalyst’s effectiveness. These findings highlight the critical role of calcination temperature and BA content in tuning the acidity and catalytic performance of BZ, where 400 °C and 25wt.% BA emerge as the most suitable condition to preserve high surface acidity and maintain a well-dispersed active phase, ultimately enhancing the catalyst’s performance in acid-catalyzed reactions.

**Table 2:** Effect of BO_3_^3−^ content and calcination temperatures on surface acidity and catalytic activity of ZrO_2_ catalysts.

Sample	S_BET_ (m^2^/g)	Pore volume (cm^3^/g)	Pore diameter (nm)	No. of acid sites/g ×10^− 19^	Ei (mV)	No. of Brønsted acid sites/g x10^− 19^	No. of Lewis acid sites/g x10^− 19^	Hydroquinone diacetate, (wt%)
ZrO_2_	17.23	0.09	3.29	6.5	74.0	--	--	Traces
5BZ-I	55.68	0.14	3.12	10.6	339.5	4.2	6.1	35.4
15BZ-I	120.16	0.17	2.73	13.9	456.3	4.9	7.3	88.5
25BZ-I	145.17	0.18	2.69	15.4	520.2	6.8	8.5	95.7
35BZ-I	103.12	0.15	2.58	13.1	436.5	5.9	7.4	52.4
50BZ-I	65.86	0.13	2.48	9.5	297.6	3.9	5.6	38.6
25BZ-II	53.42	0.15	3.42	13.5	442.2	5.1	7.4	52.7
25BZ-III	46.15	0.14	3.81	8.8	270.2	3.7	5.1	40.6

#### Pyridine adsorption

**Fig. 9:**
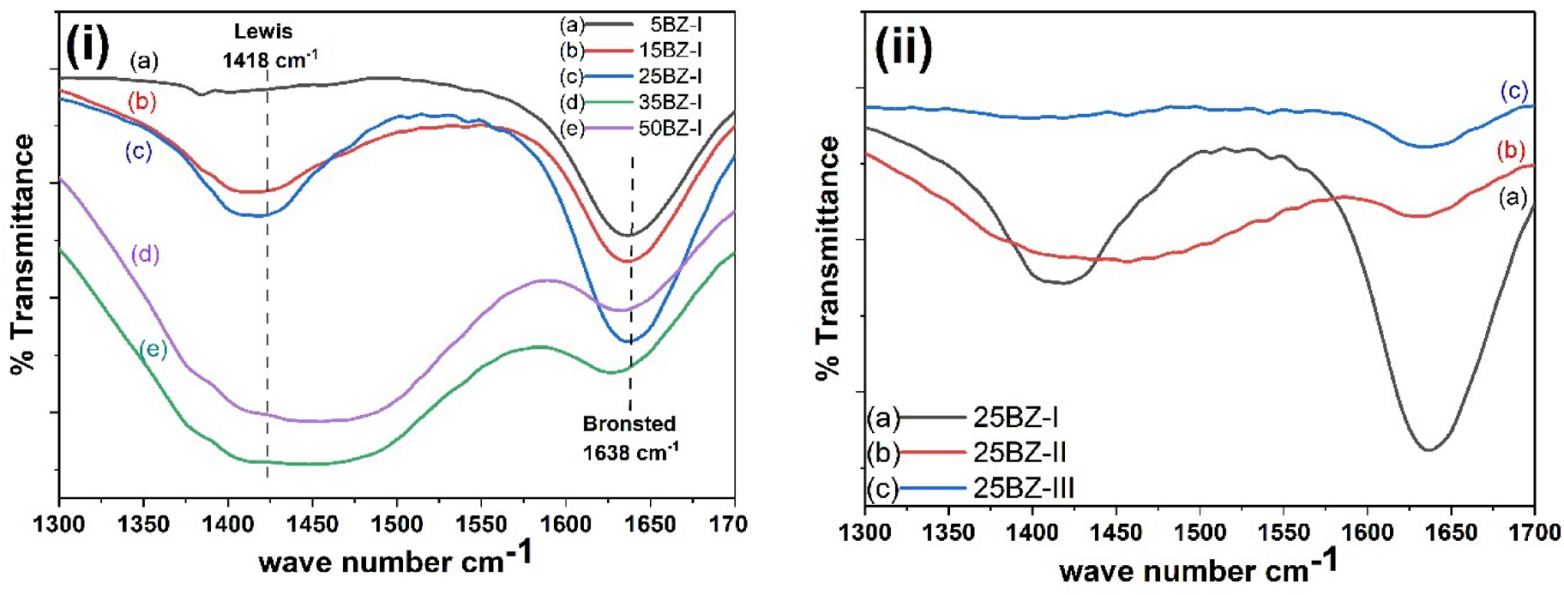
FT-IR spectra of chemisorbed pyridine on (**i**) effect of different BA content on the acidity of ZrO_2_, and (**ii**) effect of different calcination temperature on 25BZ catalyst.

The FTIR spectra of chemisorbed pyridine were recorded to evaluate the nature and distribution of acid sites on the BZ catalysts, providing insights into the relative contributions of Lewis and Brønsted acidity as illustrated in Fig. 9; Table 2. The spectra revealed two characteristic absorption bands within the range of 1300–1700 cm⁻¹, specifically at 1418 cm⁻¹ and 1638 cm⁻¹, corresponding to Lewis and Brønsted acid sites, respectively. The band at 1418 cm⁻¹ is attributed to the coordination of pyridine to Lewis acid sites, which are associated with coordinatively unsaturated Zr⁴⁺ centers capable of accepting electron pairs from pyridine molecules. This confirms the presence of exposed Zr⁴⁺ species, indicating that the catalyst surface retains strong Lewis acidity even after BA incorporation and calcination. On the other hand, the absorption band at 1638 cm⁻¹ signifies the presence of Brønsted acid sites, which originate from the interaction of pyridine with surface hydroxyl groups, leading to the formation of pyridinium ions. These findings suggest that the BZ catalysts possess both Lewis and Brønsted acid sites, with their relative abundance being dependent on the BO₃³⁻ content, as listed in Table 2 and illustrated in Fig. 9i. It is observed that as BO₃³⁻ loading increases, both Brønsted and Lewis acid sites increase, reaching a maximum at 25 wt% BO₃³⁻ loading. However, beyond this threshold, a decline in both acid site densities is observed, likely due to the agglomeration of BO₃³⁻ species, which reduces the accessibility of active sites. Furthermore, the effect of calcination temperature on acidity was investigated, and the results indicate that for the 25BZ-I catalyst, both Lewis and Brønsted acid sites decrease gradually with increasing calcination temperature from 400 °C to 600 °C, as shown in Table 2; Fig. 9ii. This reduction in acidity at higher temperatures is attributed to structural rearrangements, surface sintering, or possible dehydroxylation, which diminish the number of available active sites. The presence of well-defined Lewis and Brønsted acid sites in the BZ catalysts highlights their bifunctional nature, which is essential for catalytic applications where acidity plays a crucial role. The optimized catalyst composition and calcination conditions ensure an ideal balance between Lewis and Brønsted acid sites, making the BZ system a promising candidate for acid-catalyzed reactions.

### Catalytic activity measurement (Synthesis of 1,4-diacetoxybenzene)

#### Effect of molar ratio

The impact of the molar ratio on the synthesis of 1,4-Diacetoxybenzene over 25BZ-I was thoroughly examined to determine the most favourable reaction conditions. The results revealed that at a hydroquinone to acetic anhydride molar ratio of 1:1, only trace amounts of the product were detected, indicating that this ratio is insufficient to drive the reaction forward. However, as the molar ratio was gradually increased to 1:2, 1:3, and 1:4, a significant improvement in the product yield was observed, reaching 37.03%, 67.02%, and 95.7%, respectively. This trend suggests that a higher concentration of acetic anhydride facilitates the acetylation of hydroquinone, thereby enhancing the formation of 1,4-Diacetoxybenzene. Nevertheless, when the molar ratio was further increased to 1:5, a slight decline in yield was noted, dropping to 79.12%. This decrease may be attributed to an excess of acetic anhydride, which could potentially lead to side reactions, catalyst deactivation, or the formation of by-products that interfere with the efficiency of the process. Based on these findings, hydroquinone to acetic anhydride molar ratio of 1:4 is the most optimal for maximizing product yield while minimizing unwanted reactions, making it the preferred ratio for achieving efficient synthesis under the given experimental conditions.

#### Effect of BA content on the product yield

**Fig. 10:**
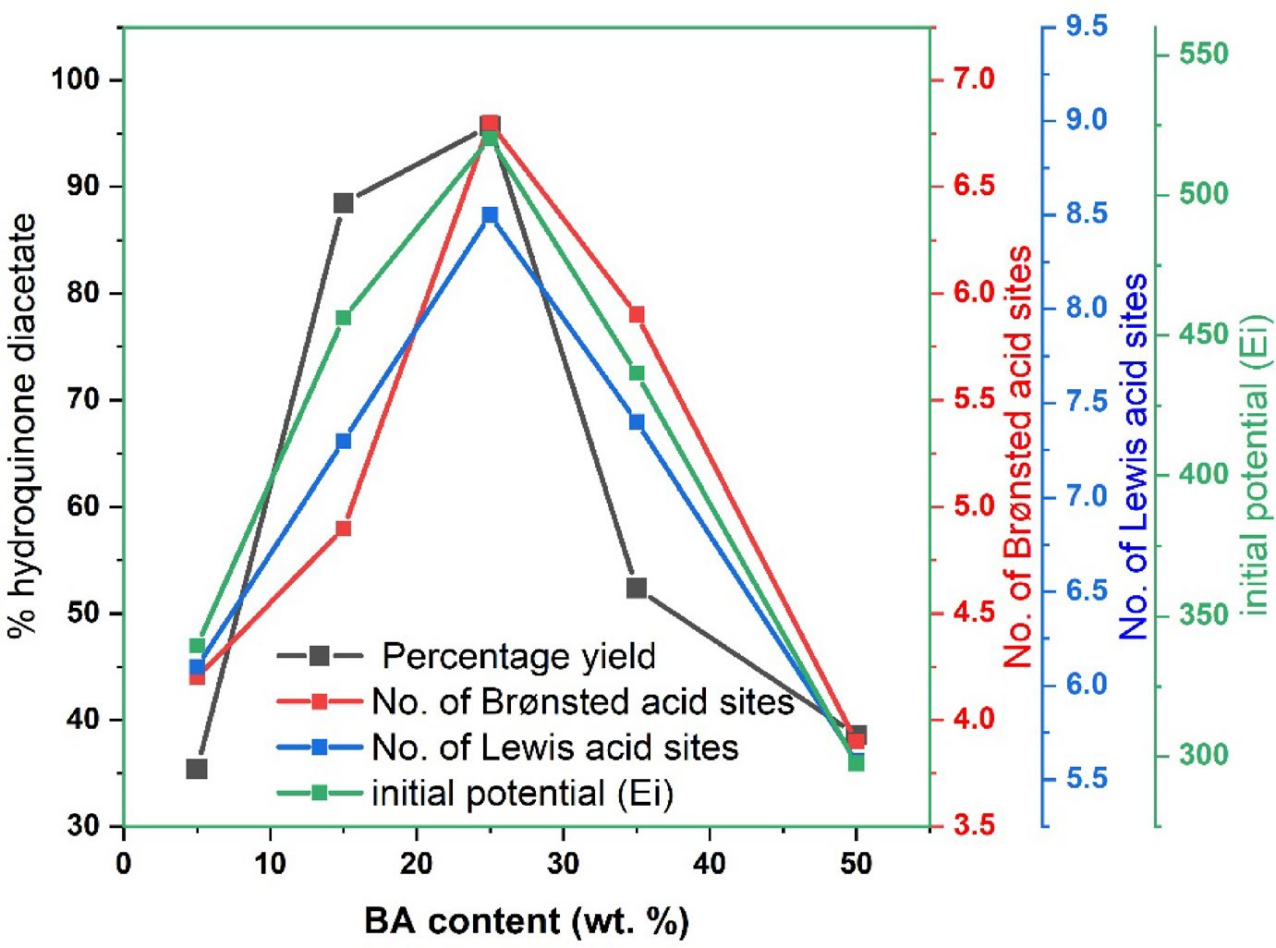
Effect of BO_3_^3−^ content on % 1,4-Diacetoxybenzene, Brønsted acid sites, Lewis acid sites and acid strength (E_i_) for BZ catalysts calcined at 400 °C.

The catalytic performance of BZ catalysts is highly dependent on the BA content, as it directly influences the total acidity, acid strength (Ei), and the nature of acid sites present on the catalyst surface. The data presented in Table 2; Fig. 10 demonstrate that as the BA content increases from 5BZ-I to 25BZ-I, there is a significant enhancement in both the number of total acid sites and the specific contributions of Brønsted and Lewis acid sites, which directly correlate with improved catalytic activity for the acetylation of hydroquinone to 1,4-diacetoxybenzene. The number of acid sites increased from 10.6 × 10⁻¹⁹ sites/g in 5BZ-I to 15.4 × 10⁻¹⁹ sites/g in 25BZ-I, while the corresponding acid strength (Ei) increased from + 339.5 mV to + 520.2 mV, indicating a substantial enhancement in surface acidity and acid strength. This increase in both Brønsted and Lewis acid sites is crucial, as both play a synergistic role in facilitating the acetylation reaction. Brønsted acid sites provide protons that activate hydroquinone molecules for nucleophilic attack, while Lewis acid sites coordinate with acetic anhydride molecules, thereby enhancing the electrophilicity of the acetylating agent and improving the overall reaction efficiency. The highest catalytic activity was observed for 25BZ-I, which exhibited the greatest number of Brønsted (6.8 × 10⁻¹⁹ sites/g) and Lewis (8.5 × 10⁻¹⁹ sites/g) acid sites, leading to an excellent hydroquinone diacetate yield of 95.7 wt%. The superior catalytic performance of 25BZ-I can be attributed to the optimal balance between Brønsted and Lewis acid sites, ensuring efficient activation of reactants and a high degree of selectivity towards the desired product. However, beyond this optimal BA content, a decline in catalytic efficiency was observed, as evidenced by the decrease in hydroquinone diacetate yield for samples with higher BA loadings, such as 35BZ-I and 50BZ-I. Specifically, when the BA content increased to 35BZ-I, the number of total acid sites decreased to 13.1 × 10⁻¹⁹ sites/g, with a corresponding reduction in Ei to + 436.5 mV, resulting in a significant drop in catalytic activity, with the hydroquinone diacetate yield decreasing to 52.4 wt%. A further increase in BA content to 50 wt% (50BZ-I) led to an even greater decline in acidity, with the number of total acid sites reducing to 9.5 × 10⁻¹⁹ sites/g and an Ei value of + 297.6 mV, ultimately yielding only 38.6 wt% of hydroquinone diacetate. The decline in catalytic performance at high BA loadings can be attributed to the agglomeration of BO₃³⁻ species on the surface of ZrO₂, which results in a reduction of accessible acid sites. Excessive BA content likely leads to the formation of large BO₃³⁻ clusters that cover active sites, hindering their participation in the reaction. This agglomeration effect reduces both Brønsted and Lewis acid site densities, thereby weakening the catalytic activity. Therefore, the results clearly indicate that 25 wt% BA loading is the most effective composition for achieving the highest catalytic activity, as it maintains an optimal balance between Brønsted and Lewis acid sites while preserving strong interactions between BO₃³⁻ species and the ZrO₂ support. This optimized acidity profile is essential for maintaining high conversion rates and selectivity, making 25BZ-I the most promising catalyst for the acetylation of hydroquinone to 1,4-diacetoxybenzene.

#### Effect of calcination temperature on the product yield

**Fig. 11:**
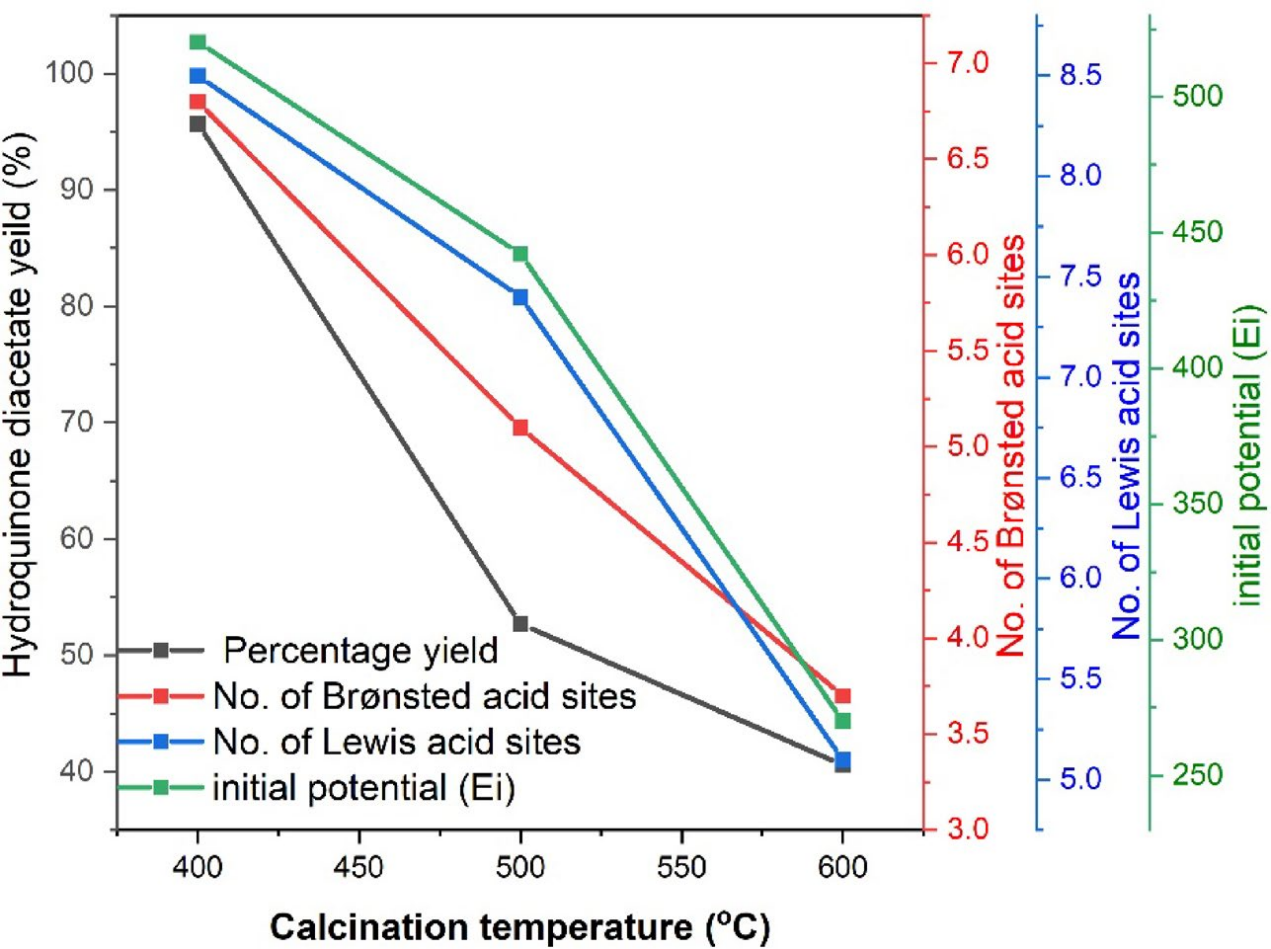
Effect of calcination temperature on % 1,4-Diacetoxybenzene, Brønsted acid sites, Lewis acid sites and acid strength (E_i_) for BZ catalysts calcined at 400 °C.

The calcination temperature plays a crucial role in determining the catalytic activity of BZ catalysts by influencing the total number of acid sites, acid strength, and structural stability. The data in Table 2; Fig. 11 indicate that the sample calcined at 400 °C (25BZ-I) exhibited the highest catalytic performance, yielding 95.7 wt% hydroquinone diacetate. This is attributed to its highest total acidity (15.4 × 10⁻¹⁹ sites/g) and strong acid strength (Ei = + 520.2 mV), along with an optimal distribution of Brønsted (6.8 × 10⁻¹⁹ sites/g) and Lewis (8.5 × 10⁻¹⁹ sites/g) acid sites. However, increasing the calcination temperature to 500 °C (25BZ-II) and 600 °C (25BZ-III) led to a decline in acidity and catalytic efficiency, with yields dropping to 52.7 wt% and 40.6 wt%, respectively. This reduction is likely due to BO₃³⁻ species agglomeration, surface sintering, and phase transformations in ZrO₂, resulting in a loss of accessible acid sites. Higher calcination temperatures also decrease the acid strength (Ei), weakening the catalytic activity. These findings suggest that 400 °C is the optimal calcination temperature to balance structural stability with active acidic sites for the highest catalytic performance.

#### Reusability test

**Fig. 12:**
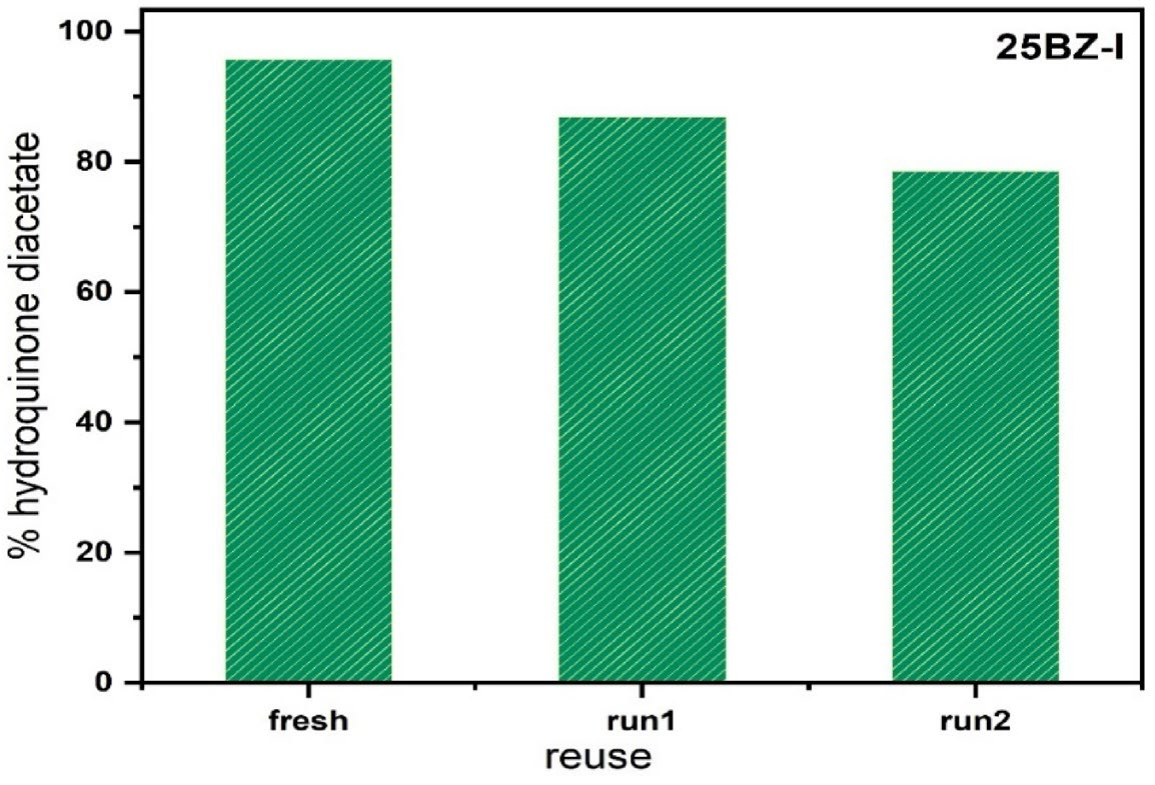
Effect of reuse of catalyst 25BZ-I on the % hydroquinone diacetate.

**Fig. 13:**
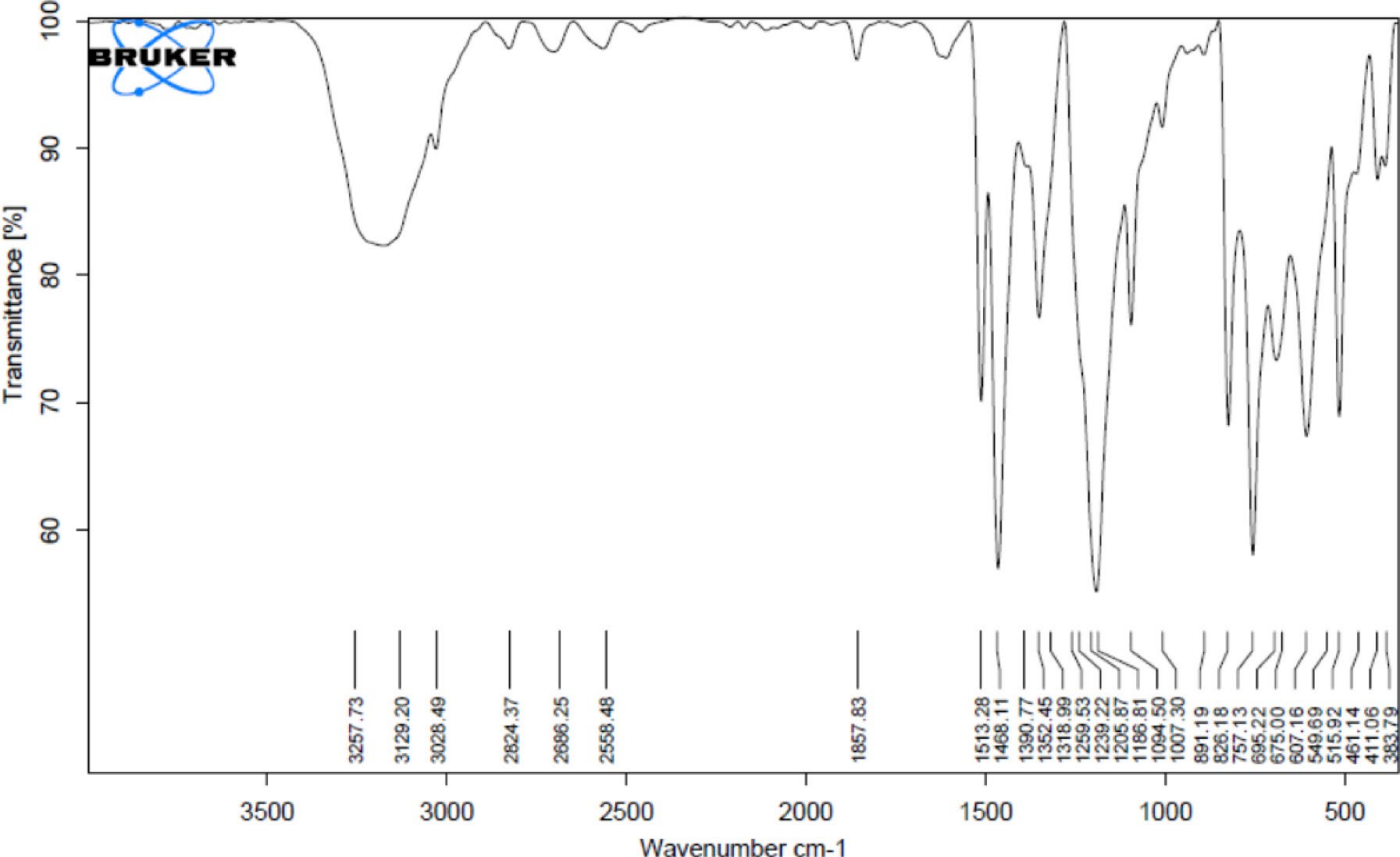
FTIR spectra of hydroquinone diacetate.

The reusability of the 25BZ-I catalyst was investigated over three consecutive reaction cycles to assess its stability and longevity in the acetylation of hydroquinone. After each reaction cycle, the catalyst was recovered through filtration, washed thoroughly with acetone to remove any adsorbed reactants or byproducts, dried, and then subjected to calcination at 400 °C for 2 h to restore its surface acidity. The catalytic performance was evaluated in each cycle, and a gradual decline in hydroquinone diacetate yield was observed. In the first cycle, the catalyst exhibited its highest activity, yielding 95.7 wt% hydroquinone diacetate. However, upon reuse in the second cycle, the yield dropped to 86.9 wt%, indicating a partial loss of catalytic efficiency. In the third cycle, the yield further declined to 78.6 wt%, suggesting a progressive deactivation of the catalyst over multiple uses. This reduction in catalytic performance can be attributed to several factors, including the partial loss of active BO₃³⁻ species, structural modifications in the catalyst due to repeated exposure to reaction conditions, and potential coke deposition or surface contamination. Despite this decline, the catalyst still retained a substantial portion of its activity, demonstrating reasonable stability and reusability. The results indicate that while the catalyst is capable of multiple uses, its long-term efficiency could be further improved through additional regeneration strategies, such as prolonged calcination, acid treatment, or optimized washing procedures to better restore its active sites. These findings highlight the importance of catalyst stability in practical applications and suggest that 25BZ-I remains an effective and reusable catalyst with proper regeneration, as illustrated in Fig. 12. The structure of the synthesized hydroquinone diacetate was confirmed by FT-IR spectroscopy and melting point analysis. The FT-IR spectrum (Fig. 13) exhibited a strong carbonyl stretching vibration at 1758 cm⁻¹, which is characteristic of the ester C = O group. Aromatic ring vibrations appeared at 1513 cm⁻¹ and 1437 cm⁻¹, while methyl C–H bending was identified at 1390 cm⁻¹. The C–O–C stretching vibrations of the ester moieties were observed at 1237 cm⁻¹, 1226 cm⁻¹, and 1187 cm⁻¹. Peaks related to aromatic and aliphatic C–H stretching were present in the 3100–2800 cm⁻¹ region, including bands at 3258 cm⁻¹, 3129 cm⁻¹, 3082 cm⁻¹, and 2924 cm⁻¹. A broad absorption in this region may also reflect residual hydroxyl stretching due to adsorbed moisture. The melting point of the product was recorded as 121–122 °C, in agreement with literature values for 1,4-diacetoxybenzene, thus confirming the product’s identity and purity.

To highlight the catalytic superiority of the optimized borated mesoporous zirconia catalyst (25BZ-I), its performance was compared with commercial zirconia and other reported mesoporous solid acid catalysts. The results (Table 3) show that 25BZ-I achieved the highest yield (95.7%) for hydroquinone acetylation under mild, solvent-free conditions, while commercial ZrO₂ exhibited only trace activity due to its low surface area and acidity. Compared to previously reported catalysts such as H₃PW₁₂O₄₀/SnO₂ and PMA/MCM-41, 25BZ-I demonstrated superior catalytic efficiency, higher surface acidity, and better reusability over three cycles^5,10,44^. This enhanced performance is attributed to the synergistic effects of boron incorporation and mesoporosity, which together promote effective reactant activation, diffusion, and acid site accessibility.

**Table 3:** Comparison of catalytic performance for hydroquinone acetylation.

Catalyst material	Catalyst quantity (g)	Temperature (°C)	Yield (%)	Reference
25BZ-I	0.015	80	95.7	This work
Commercial ZrO₂	0.015	80	Traces	This work
H₃PW₁₂O₄₀/SnO₂	0.015	80	89.2	^10^
PMA@MCM-41	0.015	90	88.4	^5^
PMA@SnO₂	0.020	80	85.5	^44^

#### Tentative mechanism and role of boron and mesoporosity

**Fig. 14:**
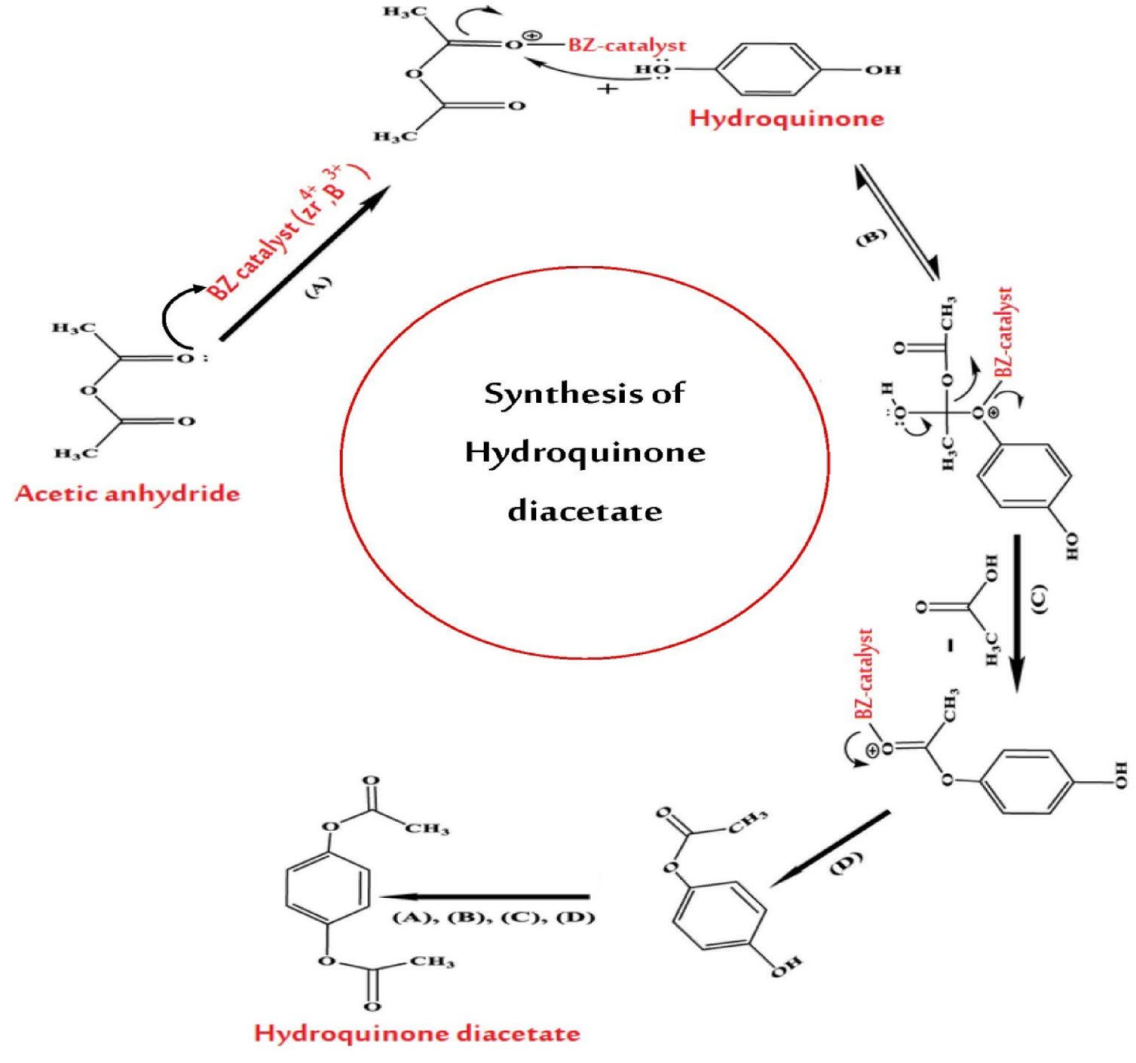
Mechanism of hydroquinone acetylation over borated mesoporous zirconia catalyst.

The acetylation of hydroquinone using acetic anhydride in the presence of borated mesoporous zirconia (BZ catalyst) is an acid-catalyzed process in which both Brønsted and Lewis acid sites synergistically enhance the reactivity and selectivity of the reaction. As illustrated in the mechanism in Fig. 14, the process begins with the activation of acetic anhydride (Ac₂O) by the Lewis acidic sites present on the BZ catalyst surface, such as coordinatively unsaturated Zr⁴⁺ and B³⁺ centers. These sites coordinate with the carbonyl oxygen of acetic anhydride, thereby polarizing the carbonyl bond and increasing the electrophilic character of the carbon atom. Concurrently, Brønsted acid sites on the catalyst, such as surface hydroxyls or boron hydroxyls, may protonate the acetic anhydride, forming an acylium ion-like intermediate that is even more susceptible to nucleophilic attack^10,13^. The activated acetic anhydride species then undergoes nucleophilic attack by one of the hydroxyl groups of hydroquinone, forming a tetrahedral intermediate that collapses to release acetic acid and yield monoacetylated hydroquinone. In a subsequent step, the remaining hydroxyl group of hydroquinone undergoes a similar acetylation reaction with another activated acetic anhydride molecule, resulting in the formation of the final product, 1,4-diacetoxybenzene (hydroquinone diacetate), completing the catalytic cycle with the regeneration of the active BZ surface sites.

## Conclusion

The successful synthesis and characterization of borated mesoporous zirconia nanoparticles have demonstrated their efficiency as a robust solid acid catalyst for the high-yield production of 1,4-diacetoxybenzene through a green and sustainable acetylation process. The catalyst’s mesoporous structure, confirmed through TEM and BET analysis, provided a high surface area of 145 m²/g, facilitating improved accessibility of reactants to active sites and resulting in enhanced catalytic performance. Under optimized reaction conditions, the catalyst achieved an impressive 95.7% yield within 90 min at 80 °C, significantly outperforming conventional acid catalysts in terms of reaction efficiency and environmental benefits. The reusability study indicated that the catalyst retained over 78% of its initial activity after three successive reaction cycles, demonstrating its stability and minimal leaching of active components. Unlike homogeneous acid catalysts, which contribute to environmental pollution and require extensive purification processes, the borated mesoporous zirconia system provided an easily separable and recyclable alternative, reducing both operational costs and hazardous waste production. Furthermore, its enhanced acidity due to boric acid modification played a critical role in improving catalytic activity and selectivity, making it a superior choice for industrial applications. These findings reinforce the significance of mesoporous solid acid catalysts in advancing sustainable chemistry, paving the way for greener and more efficient chemical processes. The study highlights the potential of borated mesoporous zirconia as a promising candidate for large-scale applications in the pharmaceutical and fine chemical industries, emphasizing its role in reducing environmental impact while maintaining high catalytic efficiency.

## Data Availability

The authors confirm that the data supporting the findings of this study are available from the corresponding author Reda S. Salama upon request (via the Email: Reda.salama@deltauniv.edu.eg), without any restrictions.
